# Wound healing and photodynamic potential of *Adiantum capillus-veneris* L. (Pteridaceae): an evaluation of the cellular effects and molecular insights

**DOI:** 10.3389/fphar.2025.1729572

**Published:** 2026-01-05

**Authors:** Linru Mou, Banaz Jalil, Francesca Scotti, Majella E. Lane, Bernd L. Fiebich, Lu Sun, Caroline Maake, Michael Heinrich

**Affiliations:** 1 Pharmacognosy and Phytotherapy, UCL School of Pharmacy, University College London, London, United Kingdom; 2 Department of Pharmaceutics, UCL School of Pharmacy, University College London, London, United Kingdom; 3 Neurochemistry and Neuroimmunology Research Group, Department of Psychiatry and Psychotherapy, Medical Center, Faculty of Medicine, University of Freiburg, Freiburg, Germany; 4 Departmental of Clinical Laboratory Center, Beijing Children’s Hospital, Capital Medical University, National Center for Children’s Health, Beijing, China; 5 Institute of Anatomy, University of Zurich, Zurich, Switzerland; 6 Department of Pharmaceutical Sciences and Chinese Medicine Resources, Chinese Medicine Research Center, College of Chinese Medicine, China Medical University, Taichung, Taiwan

**Keywords:** traditional medicinal plants, *Adiantum capillus-veneris L.*, wound healing, skin, dermatology, photodynamic therapy (PDT), HaCaT

## Abstract

**Introduction:**

Chronic and infected wounds represent a persistent global health burden. Medicinal plants offer a promising source of wound-healing agents due to their multitarget activities, long history of traditional use, and accessibility. *Adiantum capillus-veneris* L. (ACV), traditionally used to treat a range of ailments, such as respiratory, urinary, and skin disorders, was investigated for its *in vitro* wound-healing potential following methanol extraction.

**Methods:**

We evaluated the effects of methanol extracts of ACV (ACVM) on cell proliferation, migration and antioxidative capacity in human keratinocytes (HaCaT), and anti-inflammatory activity in RAW 264.7 cells. We also explored its combination with visible light phototherapy.

**Results:**

Chemical profiling via HPTLC analysis, UV/Vis spectrophotometry and HPLC analysis, together confirmed that ACVM contained more metabolites than other extracts, yielding five visible-light absorption peaks and identifying rutin and chlorogenic acid as major metabolites. At ≤100 μg/mL, ACVM was non-toxic to HaCaT cells in the absence of visible light. However, phototoxicity was evident at 200 μg/mL. ACVM (50 μg/mL) significantly promoted HaCaT migration, with a further enhancement upon exposure to light. ACVM also suppressed H_2_O_2_-induced ROS generation in a dose-dependent manner (≤50 μg/mL), while light exerted a bidirectional modulatory effect. Additionally, ACVM markedly inhibited LPS-induced secretion of CXCL2, CCL2, CXCL10, TNF-α, and IL-6 in RAW 264.7 macrophages, with effects evident at concentrations as low as 0.1 μg/mL.

**Discussion:**

These findings suggest that ACVM, particularly in combination with light-assisted therapy, shows promise for accelerating wound healing.

## 1 Introduction

Wounds are defined as any disruption in the integrity of the skin, and typically progress through the overlapping phases of haemostasis, inflammation, proliferation and remodeling (Ennis and Hill, 2016; Yazarlu et al., 2021). Disturbances caused by systematic disease or infection may impair these processes, resulting in delayed healing, chronic wounds or excessive scarring (Tabriz and Douroumis, 2022). The clinical and economic burden is considerable, particularly in regions with limited access to biomedical treatments, where traditional medicinal plants remain an essential component of primary healthcare. In 2018, the global cost of wound care was estimated at USD 18.4 billion, with projections reaching USD 25.04 billion by 2026, driven by an annual growth rate of 3.9% (Monika et al., 2022). In response, international health bodies (WHO) have increasingly emphasized the need for scientific evidence to support the safe and effective use of traditional medicines (Hoenders et al., 2024; World Health Organization, 2025).

*Adiantum capillus-veneris* L. (Pteridaceae) (ACV) is a widely distributed fern used in various traditional medical systems and valued for treating internal disorders as well as skin conditions, including wounds (Ansari and Ekhlasi-Kazaj, 2012; Qadir et al., 2025). Phytochemical studies report terpenoids, flavonoids and other phenolics as major constituents, and may account for its reported antimicrobial, antioxidant and anti-inflammatory activities (Dehdari and Hajimehdipoor, 2018; Rastogi et al., 2018; Jaafer et al., 2025). Compounds such as chlorogenic acid and rutin, which are abundant in ACV, exhibit well-established wound-healing, anti-inflammatory and antioxidant properties (Patel and Patel, 2019; Nguyen et al., 2024). Although their classification as pan-assay interfering substances (PAINS) necessitates cautious interpretation of *in vitro* data (Heinrich et al., 2020; Heinrich et al., 2022).

Despite its ethnomedicinal relevance, the wound-healing effects of ACV have been only partially explored. Aqueous extracts enhanced fibroblast survival under oxidative stress, stimulated endothelial tube formation and showed low irritation potential in the chorion allantois membrane (CAM) model (Nilforoushzadeh et al., 2014). Methanolic extracts have been shown to increase Transforming Growth Factor Beta 1 (Tgfβ1) and Vascular Endothelial Growth Factor A (Vegf-A) gene expression in fibroblasts (Negahdari et al., 2017), and ethanolic extracts demonstrated antioxidant activity in DPPH, ABTS and FRAP free radical scavenging models (Osman Mahmud et al., 2023).

*In vivo*, an ACV-based formulation also including an extract from aloe and myrrha accelerates full-thickness wound closure in male Wistar rats, downregulating Mmp9 and modulating Tgfb1, Mmp3, IL-6, and TNF-α expression in wound tissue (Galehdari et al., 2016). ACV flavonoids mitigated CCl_4_-induced hepatic oxidative damage in mice by lowering MDA and restoring SOD, CAT and GSH levels (Jiang et al., 2011). Ethanolic ACV extracts also produced anticonvulsant and analgesic effects in mice, verified in pentylenetetrazole, maximal electroshock, hot-plate and tail-immersion tests (Jain et al., 2014). The 50% ethanolic extract of ACV demonstrated anti-inflammatory effects in a carbendazim-induced hepatotoxicity model in rats by suppressing NF-κB activation and reducing TNF-α and IL-6 levels (Seif et al., 2023).

However, evidence regarding ACV’s effects on keratinocytes that play a central role in wound healing is limited. The only study involving HaCaT cells assessed antibacterial activity and evaluated cytotoxicity at a single concentration, without a full concentration–response profile or detailed statistical analysis (Khan et al., 2018). Importantly, no study has considered whether phytochemicals in ACV may exert light-dependent biological effects.

Photosensitizers can absorb photons of a specific wavelength, resulting in excitation. In the presence of oxygen, such light-activated photosensitizers generate singlet oxygen and/or Reactive Oxygen Species (ROS), which may act as mediators of various downstream biological effects (Wang et al., 2021) (see a detailed schematic illustration of the Photodynamic therapy (PDT) mechanism in the Supplementary Figure S1).

In clinical practice, while high ROS production underpins PDT for antimicrobial or anticancer applications (Papa et al., 2025), low levels of ROS generated under mild illumination can promote repair-related processes (Vallejo et al., 2020). Such low-dose photodynamic effects have been shown to enhance fibroblast and epidermal stem cell migration, support angiogenesis, regulate inflammation, and improve wound closure *in vitro*, *in vivo*, and in clinical settings (Vallejo et al., 2020; Scotti et al., 2021; Zhang et al., 2023; Mikziński et al., 2025).

We here provide a comprehensive study that aims to (i) further extend the scientific knowledge underlying wound-healing effects of methanolic ACV extracts (ACVM), including its effects on human keratinocyte migration and proliferation, as well as its antioxidative and anti-inflammatory activities, and (ii) evaluate for the first time a possible impact of light-induced effects on these processes.

Our findings strengthen the scientific understanding of a widely used ethnomedicinal species and provide insights relevant to the development of safe, evidence-based phytotherapeutic strategies for wound care, particularly in settings with limited access to medical interventions (e.g., medicines).

## 2 Materials and methods

### 2.1 Procurement of plant materials and chemicals

Authenticated dried ACV stems and leaves were sourced from Guqingtang Pharmacy (Anhui, China), with plant material harvested in August 2022 in Bozhou, Anhui Province (approximately 33.877° N, 115.770° E), China. Voucher specimens of these commercial samples (Voucher number: CN-WH-01) were deposited at the School of Pharmacy, University College London, for future reference. Full details of the purchase information of the chemicals, solvents, buffers, and culture medium used in the experiments are provided in Supplementary Table S1.

### 2.2 Plant extracts preparation

Leaves and stems of ACV were pulverized together and passed through a 60-mesh sieve. Powdered plant material (100 mg/mL) was vortexed briefly in one of the following solvents: water, ethanol, methanol, acetone or ethyl acetate, and extracted by ultrasound-assisted extraction. Briefly, the vortexed vial containing plant powder and solvents was fixed on a floating foam rack and placed into an ultrasonic bath (Grant, XUBA3, 44 KHz, 35 W) for 30 min at room temperature. The resulting supernatants were centrifuged (14,100 × g for 10 min) and filtered through a 0.45 µm PTFE syringe filter, dried by evaporation or freeze-drying, weighed, and stored at −20 °C in the dark.

### 2.3 High-performance thin layer chromatography (HPTLC)

Since the species is not included in a relevant pharmacopoeia and based on the ConPhyMP standards, a simplified protocol was used to characterize the extract (Heinrich et al., 2022). Extracts were redissolved in their original solvents to a concentration of 5 mg/mL. Aliquots (8 µL) were applied as 8 mm bands on HPTLC silica gel 60 F254 plates (Merck KGaA, Darmstadt) using a CAMAG® Linomat 5 sample applicator (CAMAG, Muttenz, Switzerland). Plates were developed using an Automatic Developing Chamber 2 (ADC 2, CAMAG) in a saturated twin-through glass chamber with a mobile phase of toluene: ethyl acetate (8:2, v/v) as described by Kumar et al. (Kumar Sagar et al., 2022). The development process involved saturation (20 min), activation (10 min), and pre-drying (5 min), all at 33% humidity. Plates were derivatized with 1% vanillin–sulfuric acid using a 022.6030 CAMAG® Derivatizer (CAMAG), and visualized under white light, 254 nm and 366 nm UV light before and after derivatization (TLC visualizer 3, CAMAG).

### 2.4 UV/Vis spectrometry

Dried acetone and methanol extracts of ACV were reconstituted in dimethyl sulfoxide (DMSO) to yield stock solutions at a concentration of 100 mg/mL. Working solutions were prepared by serial dilution in DMSO to 250 μg/mL (acetone extract) and 500 μg/mL (methanol extract). Absorbance spectra were recorded from 380 to 780 nm using a UV/Vis spectrophotometer (JENWAY 7250, Cole-Parmer, Staffordshire, UK) with DMSO serving as the blank.

### 2.5 High-performance liquid chromatography (HPLC)

Dried ACVM were reconstituted in methanol at a concentration of 1 mg/mL. Standard solutions of chlorogenic acid and rutin were prepared in methanol at concentrations of 1,000, 500, 250, 100, 50, 25, 12.5, 5, 1, and 0.1 μg/mL. All samples were filtered through 0.22 µm PTFE syringe filters before analysis.

HPLC analysis was conducted using an Agilent 1,260 Infinity II system equipped with a variable wavelength detector (Agilent Technologies, Santa Clara, CA, United States). Data acquisition and processing were performed using OpenLab ChemStation software (Agilent Technologies). Separation was achieved on an InfinityLab Poroshell 120 EC-C18 column (4.6 × 150 mm; Agilent Technologies, UK). Samples were stored in amber HPLC vials to protect from light prior to injection. HPLC conditions were adapted from Zeb and Ullah (2017) (Zeb and Ullah, 2017) with modifications for improved separation. A gradient elution was employed, with solvent A comprising 0.1% (v/v) acetic acid in water, and solvent B being methanol. The flow rate was maintained at 1 mL/min, with an injection volume of 20 µL and a column temperature of 30 °C. Detection was performed at 280 nm. The gradient profile is described in Table 1.

**TABLE 1:** Gradient elution system for *Adiantum capillus-veneris*.

ROW ID	Time (min)	Solvent A (0.1% (v/v) acetic acid in water) (%)	Solvent B (Methanol) (%)
1	5	85	15
2	25	50	50
3	30	0	100
4	60	0	100

### 2.6 Cell culture

The human immortalized keratinocyte cell line HaCaT was obtained from Caltag Medsystems Ltd. (Buckingham, UK) and cultured in Dulbecco’s Modified Eagle Medium (DMEM), supplemented with 10% FBS and 1% penicillin–streptomycin. Cells were maintained at 37 °C in a humidified incubator with 5% CO_2_.

The murine macrophage-like cell line RAW 264.7 was kindly provided by Prof. Munoz (University of Cordoba, Spain) and cultured under the same conditions as HaCaT cells.

### 2.7 Visible light phototherapy protocol and sulforhodamine B (SRB) assay

The dried ACVM obtained in Section 2.2 was re-dissolved in DMSO to prepare a 100 mg/mL stock solution, which was subsequently diluted with culture medium to the desired working concentrations prior to treatment, and this stock solution was used for subsequent tests.

HaCaT cells were seeded in 96-well plates (Corning®, CLS3596, Corning Inc., Corning, NY, United States) at a density of 1 × 10^4^ cells per well and incubated overnight to allow adherence. The cells were then exposed to fresh medium containing ACVM (diluted from stock solution) to reach the working concentrations of 1, 10, 50, 100, and 200 μg/mL for a total of 48 h under cell culture conditions. Control treatments included blank control (no treatment, medium-only), solvent control (0.2% DMSO, corresponding to the final DMSO concentration after diluting the 100 mg/mL stock solution 500-fold in DMEM) and positive control (epidermal growth factor, EGF) at 10 ng/mL. To assess the influence of visible light exposure, two identical plates were prepared. Following a 5-h pre-treatment with the plant extracts, the light exposure group was irradiated in the presence of extracts, using a LED light source (NorbSMILE; 380–780 nm, 9 W, 680 lumens, 7,450 lx) placed 15 cm above the culture surface for 30 min, after which incubation was continued until the 48 h endpoint. Another plate was kept in the dark. Both sets were then incubated for a total of 48 h under cell culture conditions in the dark.

Thereafter, cell viability was assessed using the sulforhodamine B (SRB) assay, which quantifies cell numbers by measuring total protein content. Measurements were conducted according to the method of Vichai and Kirtikara (2006) (Vichai and Kirtikara, 2006). Cells were fixed by adding 100 µL of 10% (w/v) trichloroacetic acid and incubated at 4 °C for 1 h. The plates were then washed five times with tap water and air-dried. Subsequently, 100 μL of 0.057% (w/v) SRB was added to each well and incubated at room temperature for 30 min. Unbound dye was removed by rinsing five times with 1% (v/v) acetic acid, followed by air-drying. Protein-bound dye was solubilized with 200 µL of 10 mM Tris base, and plates were shaken for 20 min. Absorbance was recorded at 515 nm using a microplate reader (Infinite M200; Tecan Group Ltd., Männedorf, Switzerland). Cell proliferation was expressed as a relative ratio calculated using the following equation:

$$\text{Relative proliferation ratio}=\frac{OD\left(treatment\right)-OD\left(blank\right)}{OD\left(control\right)-OD\left(blank\right)\;}\times 100\%$$

### 2.8 Scratch assay

The scratch assay protocol was based on Liang et al. (2007) (Liang et al., 2007). HaCaT cells were seeded in 12-well plates (Corning®, CLS356500) at a density of 2 × 10^5^ cells per well and cultured to approximately 90% confluence. To prevent proliferation-driven closure of the scratch and eliminate potential false-positive results, cells were pre-treated with 5 μg/mL mitomycin C for 2 h prior to wounding, followed by thorough washing with PBS. A linear scratch was introduced with PBS inside the well manually, using a sterile 200 µL pipette tip guided by a ruler to ensure consistency. Before wounding, three reference marks were drawn on the underside of each well to aid in image alignment. After scratching, the monolayer was gently washed with PBS to remove detached cells and debris. Cells were then treated with culture medium containing ACVM to final working concentrations of 1, 10, 50, and 100 μg/mL in each well for 24 h. Control treatments included blank control (medium-only), solvent control (0.1% DMSO, corresponding to the final DMSO concentration after diluting the 100 mg/mL stock solution 1000-fold in DMEM) and positive control (10 ng/mL EGF). For light control group, the same visible light phototherapy protocol as described in Section 2.7 assay was applied after the exposure of scratch wounds to ACVM extracts.

Images were captured at 0, 12, and 24 h post-wounding using the EVOS M5000 Imaging System (Thermo Fisher Scientific). Wound closure was quantified by analyzing the scratch area using ImageJ (version 1.53t), and the percentage of migration was calculated as follows:

$$\text{Migration rate}=\frac{Initial\;Wound\;Area-Remaining\;Wound\;Area}{Innitial\;Wound\;Area}\times 100\%$$

### 2.9 2′, 7′-Dichlorodihydrofluorescein diacetate (DCFH-DA) assay

Intracellular ROS levels were quantified using the DCFH-DA assay adapted from Kim et al. (2021) (Kim et al., 2021), and the fluorescence intensity was measured by a plate reader. This method provides quantitative data rather than microscopic images.

HaCaT cells were seeded in black 96-well plates (Corning®, CLS3631) at a density of 1 × 10^5^ cells per well and incubated overnight under cell culture conditions. Cells were then treated for 24 h with culture medium diluted ACVM stock solution at working concentrations of 1, 10, 50 and 100 μg/mL, or with 10 μg/mL l-ascorbic acid as a positive control. To investigate the effect of light, two identical plates were prepared, and one was subjected to the same visible light phototherapy protocol as described in Section 2.7. Following treatment, cells were exposed to 0.003% (∼800 μM) hydrogen peroxide (H_2_O_2_) for 1 h at 37 °C to induce oxidative stress. After stimulation, cells were washed with serum-free medium to remove residual H_2_O_2_. Subsequently, 100 μL of 10 μM DCFH-DA was added to each well and incubated for 30 min at 37 °C in the dark. Excess dye was removed by washing with serum-free medium, and 50 μL of fresh serum-free medium was added. Fluorescence intensity was measured using a microplate reader (excitation: 488 nm; emission: 525 nm; Infinite M200; Tecan Group Ltd., Männedorf, Switzerland) to quantify intracellular ROS levels. Three replicates were set for each treatment and 6 independent measurements were performed.

### 2.10 3-[4, 5-dimethylthiazol-2-yl]-2, 5 diphenyl tetrazolium bromide (MTT) assay

The MTT protocol followed standard laboratory procedure and is based on Sun et al. (2024b). RAW 264.7 cells were seeded into 96-well plates (Corning®, CLS3596) at a density of 1 × 10^5^ cells per well and incubated overnight under cell culture conditions to allow for adherence. The next day, the medium was replaced with fresh medium containing ACVM, prepared by diluting the stock solution with culture medium to final working concentrations of 0.1, 1, 10, or 50 μg/mL, and cells were incubated for 30 min. Thereafter, lipopolysaccharide (LPS; 100 ng/mL; *E. coli* O127:B8; Sigma-Aldrich, Taufkirchen, Germany) was additionally pipetted to the wells, and cells were incubated for 20 h. Subsequently, 20 μL of MTT solution (Merck KGaA, Darmstadt, Germany) was added to each well and incubated for an additional 4 h. The supernatant was carefully removed, and the resulting formazan crystals were solubilized in DMSO. Absorbance was measured at 595 nm using an MRXe microplate reader (Dynex Technologies, Denkendorf, Germany).

### 2.11 Enzyme-linked immunosorbent assay (ELISA)

ELISA was performed according to established laboratory protocol (Sun et al., 2024b), with adaptations for the different cell lines used in this study. RAW 264.7 cells were seeded in 96-well plates (Corning®, CLS3596) at a density of 1 × 10^5^ cells/well and incubated overnight. Cells were treated with ACVM diluted in culture medium to working concentrations of 0.1, 1,10 and 50 μg/mL for 30 min, after which LPS was added at a final working concentration of 100 ng/mL in each well, followed by incubation for 24 h. Culture supernatants were collected and centrifuged at 1,000 × g for 5 min at 4 °C. Concentrations of tumor necrosis factor alpha (TNF-α), interleukin 6 (IL-6), C-X-C motif chemokine ligand 10 (CXCL10), C-X-C motif chemokine ligand 2 (CXCL2), and C-C motif chemokine ligand 2 (CCL2) were measured using commercial ELISA kits (cat. nos. DY410, DY406, DY466, DY452, and DY479; Bio-Techne GmbH, Wiesbaden, Germany) that contain all necessary antibodies, standards, and solutions, according to the manufacturer’s protocols.

Briefly, 96-well plates were coated with capture antibodies and incubated overnight at 4 °C. Plates were washed and blocked, then incubated with serially diluted recombinant mouse cytokine/chemokine standards and test samples. Following additional washing steps, detection antibodies were added and incubated for 2 h, followed sequentially by streptavidin-HRP, 1x 3,3′,5,5′-tetramethylbenzidine (TMB) solution and stop solution (2 N H_2_SO_4_). Absorbance was read at 450 nm using an MRXe microplate reader (Dynex Technologies).

### 2.12 Statistical analysis

Statistical analyses were conducted using IBM SPSS Statistics for MacOS, version 29.0.0.0 (241) (IBM Corp., Armonk, NY, United States), while graphical representations were generated using GraphPad Prism for MacOS, version 9.5.0 (525) (GraphPad Software Inc., San Diego, CA, United States). Data are presented as mean ± standard deviation (SD) from a minimum of three independent biological replicates and were analyzed using one-way ANOVA. Statistical significance was defined as p < 0.05 (*), p < 0.01 (**), and p < 0.001 (***) as indicated above the corresponding bars.

## 3 Results and discussions

### 3.1 High-performance thin layer chromatographic analysis

HPTLC was utilized to assess the extraction performance of solvents with different polarities (water, ethanol, methanol, acetone, and ethyl acetate), based on their ability to extract a broad range of metabolites. The developing solvent system was Toluene: EtOAc (8:2, v/v), based on Kumar Sagar et al., 2022; Kumar Sagar et al., 2022). A total of 15 distinct bands were revealed in ACVM under all visualization conditions, followed by the ethanol and acetone extracts, each displaying 14 bands. Thirteen bands were found in the ethyl acetate extract, and only two bands were observed in the aqueous extract. The Rf values corresponding to the bands detected in each extract are summarized in Table 2. Notably, the methanol extract exhibited stronger band intensities compared to the other solvent extracts, particularly at Rf values of 0.83, 0.72, and 0.64. The HPTLC profiles under different light sources before or after derivatization are presented in the Supplementary Figures S2–S4.

**TABLE 2:** Rf value of each marked visualized band in different extracts of *Adiantum capillus-veneris*.

*A. capillus-veneris* L	Water	Ethanol	Methanol	Acetone	EtOAc
*Rf* Value		0.83	0.83	0.83	0.83
		0.80	0.80	0.80	0.80
		0.72	0.72	0.72	0.72
		0.64	0.64	0.64	0.64
		0.59	0.59	0.59	0.59
		0.55	0.55	0.55	0.55
		0.52	0.52	0.52	0.52
		0.48	0.48	0.48	0.48
		0.40	0.40	0.40	0.40
		0.24	0.24	0.24	0.24
		0.23	0.23	0.23	
		0.22	0.22	0.22	0.22
	0.16	0.16	0.16		
	0.13	0.13	0.13	0.13	0.13
			0.10	0.10	0.10

Based on HPTLC results, it was observed that polar organic solvents (e.g., ethanol and methanol) extracted a higher number of metabolites than less-polar solvents (e.g., acetone and ethyl acetate) and water. Methanol exhibited the highest extraction performance compared with other solvents, suggesting that the secondary metabolites in this plant are predominantly polar or semi-polar. Notably, water exhibited the lowest extraction yield. Although water is more polar than methanol, its high viscosity and poor permeability limit its ability to penetrate plant cells. As a result, even if certain metabolites are water-soluble, they may remain trapped within the cells, leading to a lower overall extraction yield (Basri and Nor, 2014).

### 3.2 UV/Vis spectroscopy

Methanol extracts were initially selected for UV/Vis analysis based on HPTLC results. However, considering that less polar metabolites with potential photoactive properties might not be efficiently extracted by methanol alone, the acetone extract was subsequently included as a representative of less polar solvent extracts. This approach allowed broader metabolite coverage across different polarity ranges while minimizing sample consumption.

**FIGURE 1:**
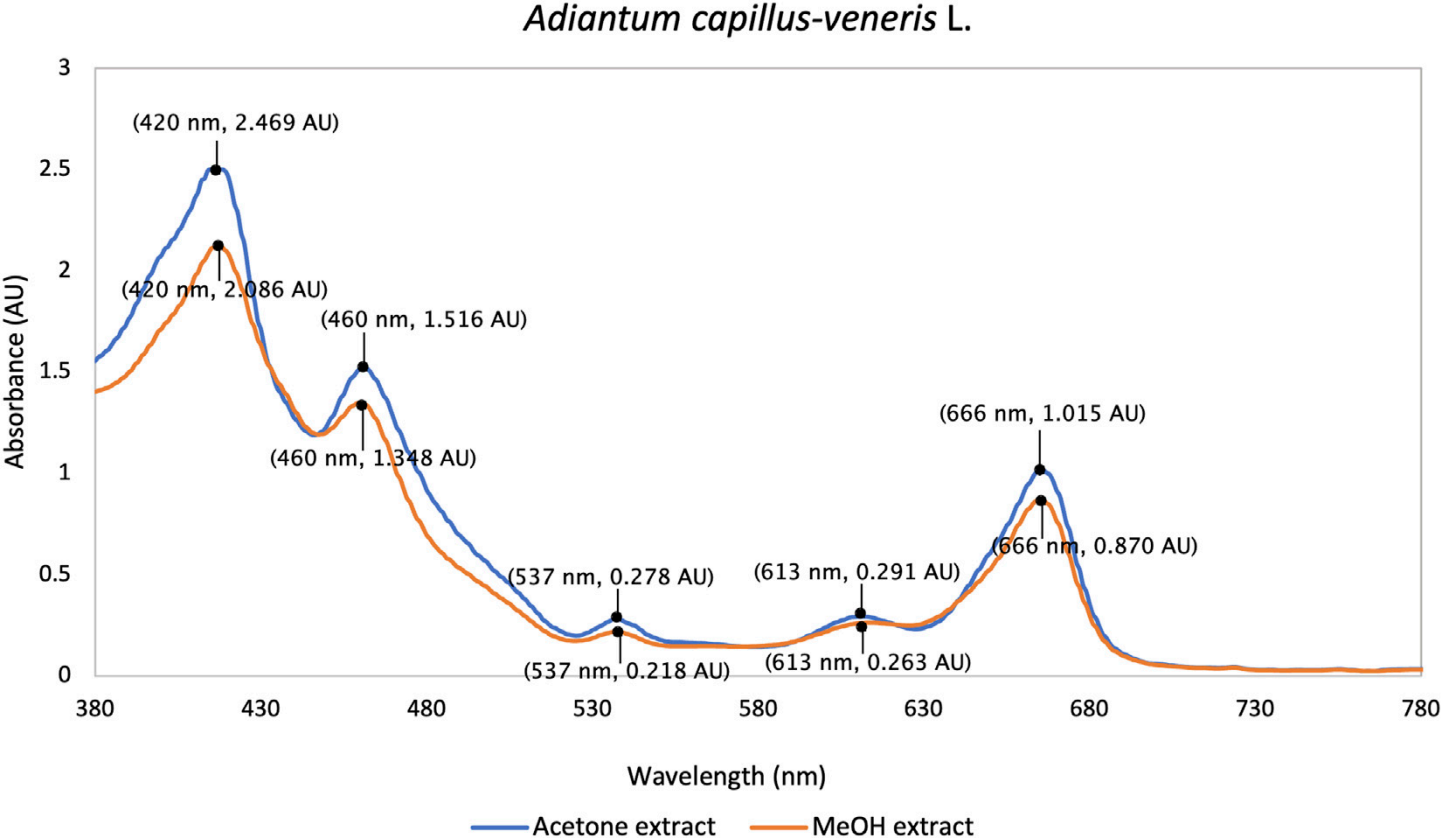
UV/Vis absorption spectra of acetone and methanol extracts of ACV in the visible light wavelength range (380 nm–780 nm).

This is the first report on visible light absorption spectra of methanol and acetone extracts of ACV. In UV/Vis spectrometry, both acetone (250 μg/mL) and methanol (500 μg/mL) extracts showed a similar pattern with a strong band of 420 nm in the Soret region, a smaller peak at 460 nm, a major Q band at 666 nm and two minor Q bands at 537 nm and 613 nm (Figure 1). This spectral fingerprint may indicate the dominant presence of porphyrin-type pigments, including chlorophyll derivatives such as pheophytin (Kang et al., 2018; Taniguchi and Lindsey, 2021). The efficient absorbance in the 600–700 nm range matches the established therapeutic window of PDT, enabling the generation of singlet oxygen or ROS under visible (red) light application at a clinically relevant tissue depth (Mallidi et al., 2016).

### 3.3 High-performance liquid chromatography-based quantification of rutin and chlorogenic acid

**FIGURE 2:**
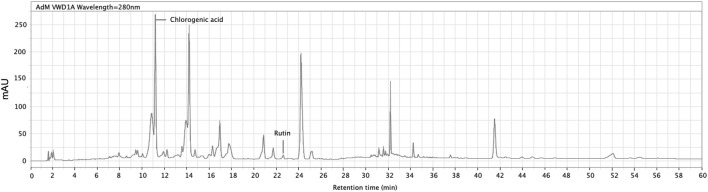
HPLC profile of crude methanol extract of *Adiantum capillus-veneris*. Peaks at retention time of 11.168 min and 22.599 min were identified as chlorogenic acid and rutin, respectively. The detection was set at 280 nm (InfinityLab Poroshell 120 EC-C18 column, 4.6 × 150 mm).

The quantification of chlorogenic acid and rutin in the ACVM was performed using an Agilent 1,260 Infinity II HPLC system, with a detection wavelength set at 280 nm. Multiple reviews report the presence of triterpenoids, flavonoids, phenylpropanoids/phenolic acids, saponins and steroids in ACV (Dehdari and Hajimehdipoor, 2018; Jaafer et al., 2025; Qadir et al., 2025). Among these, flavonoids and phenylpropanoid-type phenolic acids are particularly relevant to the present study, as they possess conjugated aromatic systems. For example, rutin (a flavonol glycoside) and chlorogenic acid (a phenylpropanoid-type phenolic acid) were selected as marker compounds due to their *in vivo* demonstrated relevance to wound repair (Almeida et al., 2012; Chen et al., 2013; Chen et al., 2020; Al-Ghanayem et al., 2024) and stable chromatographic detectability, which here provides a focused preliminary chemical characterization rather than exhaustive profiling. Their reported photoreactivity further supports their relevance to the light-assisted activity examined here (Bhattacharyya et al., 2014; Choi et al., 2014). Identification was achieved by matching the retention times of peaks in the sample with those of reference standards. The HPLC chromatogram of the methanol extract is shown in Figure 2. The retention time, peak area, and peak height of the main metabolites are summarized in Table 3. The methanolic extract was analyzed at a concentration of 1 mg/mL.

**TABLE 3:** Retention time, area, height, and area% of peaks detected in crude methanol extract of *Adiantum capillus-veneris.*

RT (min)	Area	Height	Area %
10.839	1742.7	83.6	10.64
11.168	2079.1	271.1	12.69
11.886	167.6	11.9	1.02
13.903	1,008.9	62.6	6.16
14.188	2022.7	246.9	12.35
16.278	225.6	23.7	1.38
16.932	960	70.6	5.86
17.733	662.4	28.6	4.04
20.836	545	46	3.33
21.697	231.4	21.4	1.41
22.599	75.810	7.790	0.463
24.181	2,367.1	201.7	14.45
32.172	535.7	142.5	3.27
41.484	885.1	75.5	5.4
52.053	300.8	9.5	1.84

Calibration curves were constructed using eight concentration points, spanning from 5 μg/mL to 1,000 μg/mL. The calibration curve for chlorogenic acid was described by the equation y = 19.511x+148.17, with a correlation coefficient (*R* ^2^) of 0.999. For rutin, the calibration equation was y = 8.4964x−85.257, yielding an *R* ^2^ value of 0.998. The curves were derived by plotting the peak area *versus* the known concentrations of the respective standards. Based on these calibration data, the concentrations of chlorogenic acid and rutin in the crude methanol extract (1 mg/mL) were determined to be 99.99 μg/mL and 18.96 μg/mL, respectively. The residual method was employed to determine the limits of detection (LOD) and quantification (LOQ) using the equations LOD = 3.3σ/S and LOQ = 10σ/S, where σ is the standard deviation of the residuals and S is the slope of the calibration curve. The calculated LOD and LOQ values for chlorogenic acid were 16.28 μg/mL and 49.33 μg/mL, respectively, while those for rutin were 22.53 μg/mL and 68.26 μg/mL. The measured concentration of chlorogenic acid exceeded its LOQ, indicating its relatively high abundance, whereas the concentration of rutin was below its LOD.

Although rutin was detected, its signal intensity was weak. While rutin is frequently reported as a major flavonoid constituent of ACV (Nilforoushzadeh et al., 2014; Al-Khesraji and Maulood, 2017), Al-Khesraji and Maulood (2017) demonstrated that rutin abundance can vary substantially among samples from different geographical sources (Al-Khesraji and Maulood, 2017). Together with findings from this study, suggest that variations in compound composition linked to plant origin may influence the bioactivity of ACV extracts, and their potential relevance to photodynamic effects. To better characterise the constituents contributing to light-assisted wound-healing activity, further analyses are planned using LC–MS/MS to identify more abundant metabolites in the methanol extract, particularly flavonols such as kaempferol, quercetin and their derivatives, which have been reported to exhibit anti-inflammatory, antioxidant and wound-healing activities (Qadir et al., 2025). In addition, a comparative assessment of samples from different regions will be undertaken to determine whether geographical variability in chemical profiles may affect PDT-related activity.

### 3.4 Effects of methanolic extract on keratinocyte viability

**FIGURE 3:**
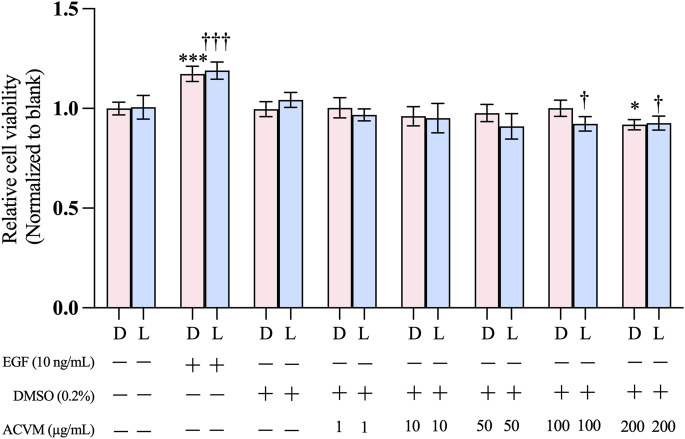
Effect of ACVM on the viability of HaCaT cells after 48 h of treatment under dark (D) conditions or 30 min light exposure (L). Cell viability was assessed using the SRB assay. Results are expressed as the mean ± SD of six independent measurements. *p* < 0.05 (*), *p* < 0.01 (**) and *p* < 0.001 (***) indicate significant differences compared to the blank group under dark condition. *p* < 0.05 (†), *p* < 0.01 (††) and *p* < 0.001 (†††) indicate significant differences compared to the blank group with 30 min light exposure.

The SRB assay was performed to exclude possible cytotoxic effects of ACVM on HaCaT cells. Under both tested conditions (without and with light), positive controls with 10 ng/mL EGF significantly enhanced cell viability, whereas solvent controls with 0.2% DMSO showed no effect compared with untreated cells (medium-only). As shown in Figure 3, in the dark, none of the ACVM concentrations tested (1–200 μg/mL) elicited a biologically relevant effect on the viability of HaCaT cells (viability at the highest concentration of 200 μg/mL: 91.91% ± 2.53% compared to the solvent control). Despite the presence of photosensitizers, exposure of ACVM-treated cells to visible light for 30 min did not reduce the cell viability below about 90% (viability at 200 μg/mL: 92.31% ± 3.63% and 100 μg/mL: 92.70% ± 3.47% compared to the untreated group) (Figure 3), indicating that unwanted photodynamic cell killing was negligible. Under the conditions used in our study, the methanolic extract can thus be considered as nontoxic and safe to use for wound healing assays, even under light exposure. In subsequent experiments, the plant extract was applied at concentrations up to 100 μg/mL.

Our data demonstrated that ACVM was well tolerated by HaCaT cells at concentrations up to 200 μg/mL. This aligns with a previous report, which showed good tolerance of ethanolic and aqueous extracts at 100 μg/mL (Khan et al., 2018). *Nilforoushzadeh et al.* reported that polar and moderately polar fractions (aqueous and butanolic) of ACVM showed no cytotoxic effect on human dermal fibroblasts up to 500 μg/mL, while cytotoxicity appeared with ethyl acetate fractions at 250 μg/mL and with hexane fractions at 500 μg/mL. Similarly, mouse fibroblasts tolerated methanolic extracts up to 1,000 μg/mL (Nilforoushzadeh et al., 2014; Negahdari et al., 2017). These discrepancies likely reflect both solvent polarity and intrinsic differences in cell-type sensitivity.

### 3.5 Effect of ACVM on the migration of HaCaT cells in scratch assays

**FIGURE 4:**
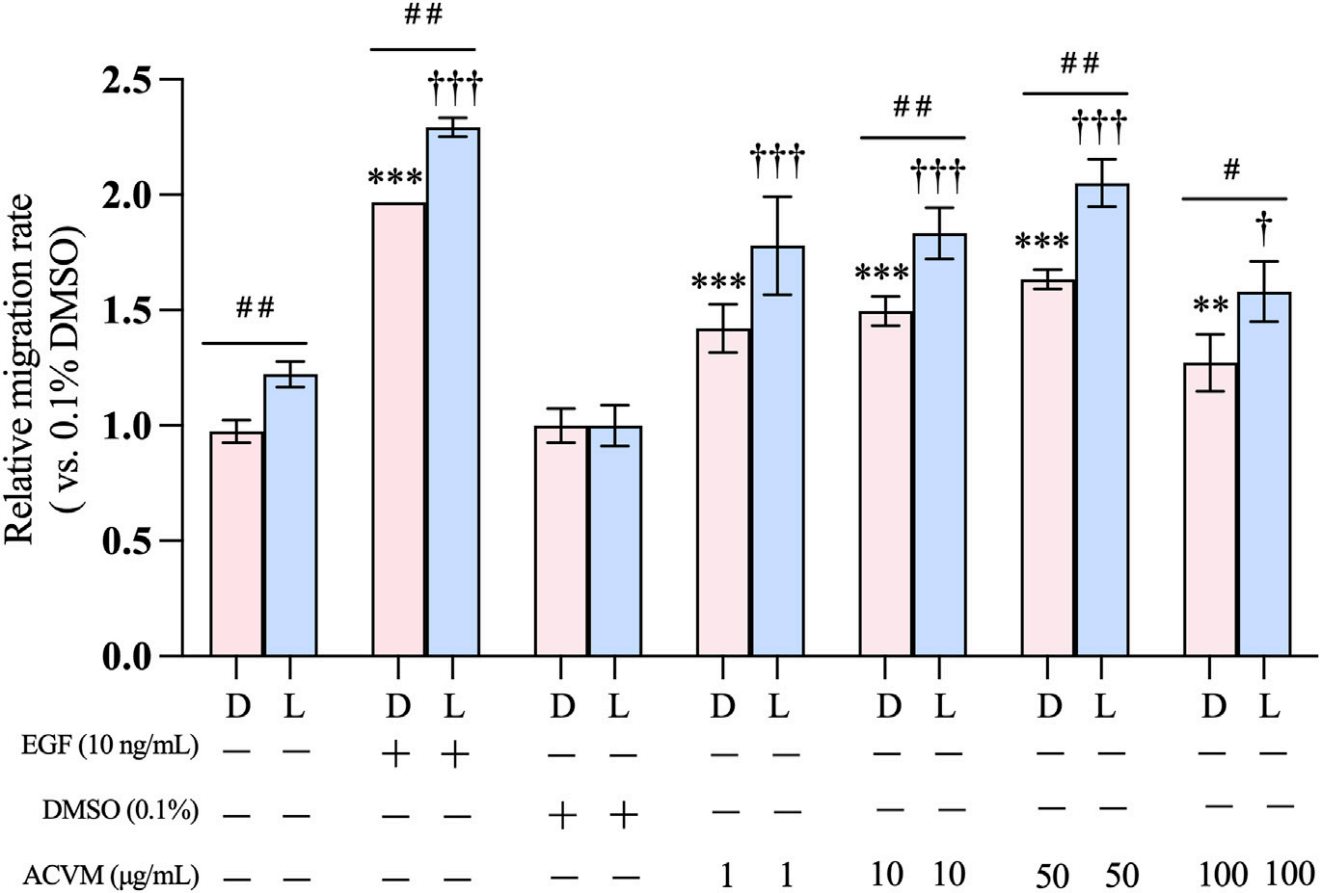
Effect of ACVM on migration rate of HaCaT cells in darkness (D) or with 30 min visible light exposure (L). Data are normalized to solvent control (0.1% DMSO) group and expressed as mean ± SD of three independent measurements. *p* < 0.05 (*), *p* < 0.01 (**) and *p* < 0.001 (***) indicate significant differences compared to the blank group under dark condition. *p* < 0.05 (†), *p* < 0.01 (††) and *p* < 0.001 (†††) indicate significant differences compared to the blank group with 30 min light exposure. *p* < 0.05 (#), *p* < 0.01 (##) and *p* < 0.001 (###) indicate significant difference between the same treatment but from two light conditions group.

To account for differences in initial wound shape and size and to assess whether light exposure itself influences migration, all data were normalized to the solvent control (0.1% DMSO) and expressed as fold changes relative to it. After 24 h of treatment (Figure 4), 0.1% DMSO alone showed no effect, whereas the positive control (10 ng/mL EGF) significantly enhanced HaCaT migration by ∼2-fold compared with the blank (medium-only).

Notably, exposure to visible light alone promoted cell migration. In the blank control, migration under darkness was 0.98 ± 0.05-fold relative to 0.1% DMSO, while light increased it to 1.22 ± 0.06-fold (*p < 0.01*). A similar light-enhanced effect was observed with ACVM treatment. In darkness, ACVM significantly increased migration in a dose-dependent manner, reaching 1.50 ± 0.06-, 1.63 ± 0.04-, and 1.27 ± 0.12-fold at 10, 50, and 100 μg/mL, respectively. With light exposure, these values rose further to 1.83 ± 0.11-, 2.05 ± 0.10-, and 1.58 ± 0.13-fold. Among the tested concentrations, 50 μg/mL ACVM showed the strongest pro-migratory effect, although the effect declined at 100 μg/mL.

This study is the first to evaluate the effect of ACV on keratinocyte migration. Consistent with our findings, Negahdari et al. (2017) reported that ACVM significantly promoted mouse dermal fibroblast migration at 50 μg/mL (Negahdari et al., 2017). Light-enhanced cell migration can be attributed to photobiomodulation, which involves the application of low-dose visible or near-infrared light to modulate cellular activity, which has been shown to promote wound healing by stimulating keratinocyte migration, proliferation, and angiogenesis (Kuffler, 2016). Sutterby et al. demonstrated that orange (610 nm, 31.12 mJ/cm^2^) and red light (660 nm, 31.36 mJ/cm^2^) significantly increased HaCaT cell migration after 48 h treatments (Sutterby et al., 2022). In our study, we applied a broad spectrum of visible light and observed results consistent with these findings. Furthermore, under identical light conditions, ACVM also enhanced cell migration, suggesting that the overall effects reflect a combination of ACVM activity and photobiomodulation. However, the mechanisms by which these two factors interact remain to be elucidated.

**FIGURE 5:**
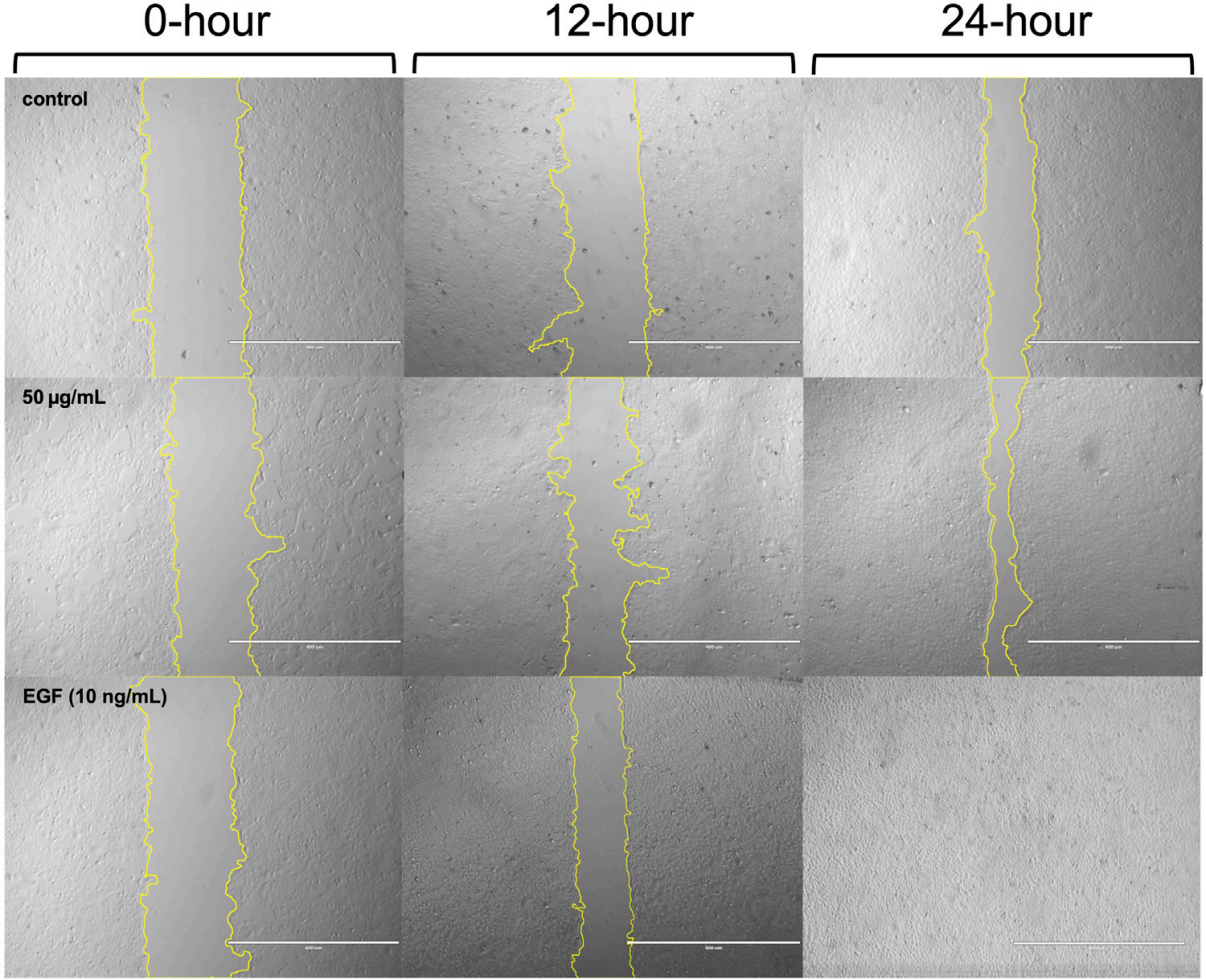
Representative phase-contrast images of HaCaT cells from the blank group, ACVM (50 μg/mL), and EGF (10 ng/mL) treatment groups at 0, 12, and 24 h under dark conditions. Wound edges were outlined using yellow lines in ImageJ, and the area between the boundaries was quantified to assess cell migration. Images are representative of three independent experiments. Scale bar = 400 µm.

**FIGURE 6:**
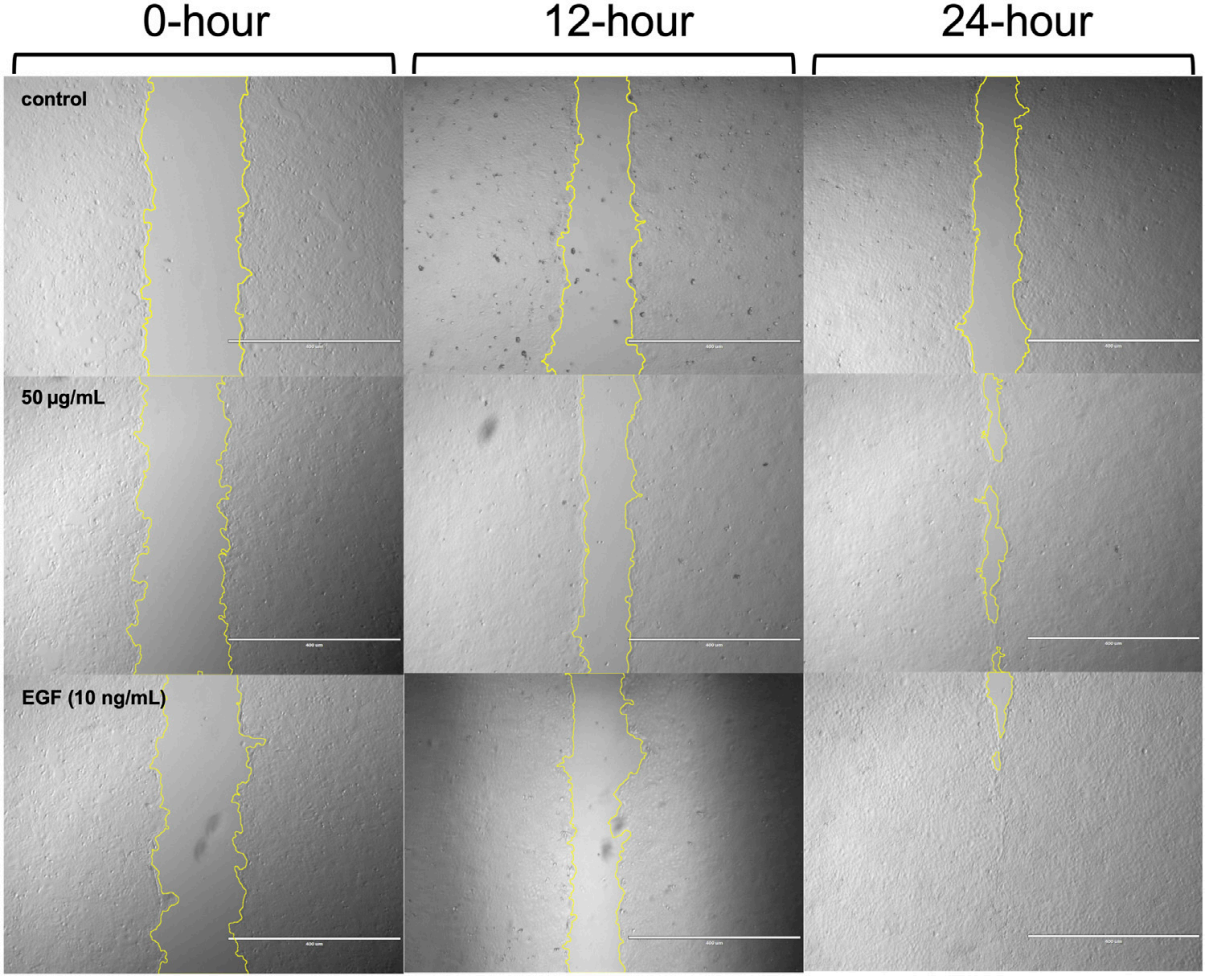
Representative phase-contrast images of HaCaT cells from the blank group, ACVM (50 μg/mL), and EGF (10 ng/mL) treatment groups at 0, 12, and 24 h with 30 min light irradiation. Wound edges were outlined using yellow lines in ImageJ, and the area between the boundaries was quantified to assess cell migration. Images are representative of three independent experiments. Scale bar = 400 µm.

Representative images of cells from the control group, as well as those treated with 50 μg/mL ACVM or 10 ng/mL EGF, were captured at 0, 12, and 24 h under dark conditions (Figure 5) and following 30 min light exposure (Figure 6).

### 3.6 Antioxidant activity of ACVM on H_2_O_2_-induced HaCaT cells

**FIGURE 7:**
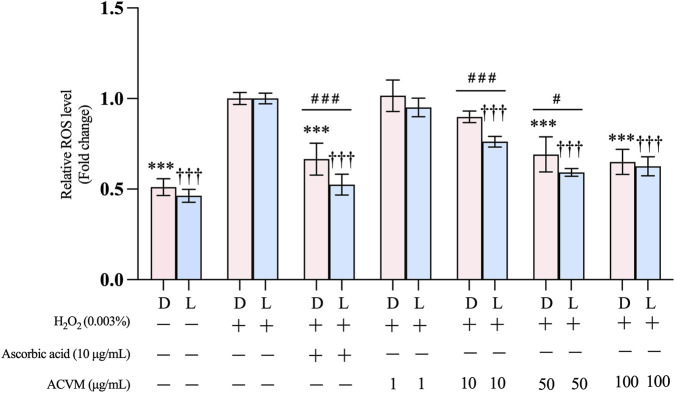
Effect of ACVM on H_2_O_2_-induced production of ROS in HaCaT cells, with 24 h treatment under dark condition (D) or 30 min light exposure (L). Results are expressed as the mean ± SD of six independent measurements. *p* < 0.05 (*), *p* < 0.01 (**) and *p* < 0.001 (***) indicate significant differences compared to the H_2_O_2_ stimulation group under dark conditions. *p* < 0.05 (†), *p* < 0.01 (††) and *p* < 0.001 (†††) indicate significant differences compared to the H_2_O_2_stimulation group with 30 min light exposure. *p* < 0.05 (#), *p* < 0.01 (##) and *p* < 0.001 (###) indicate significant difference between the same treatment but from two light conditions group. Data was normalized to the H_2_O_2_ stimulation group, which was set as 1 and presented as the fold change.

The effect of ACVM on H_2_O_2_-induced oxidative stress in HaCaT cells was evaluated using the DCFH-DA probe as a marker for ROS. In both the absence and presence of light (Figure 7), exposure to H_2_O_2_ resulted in a significant 1.96 ± 0.07-fold and 2.16 ± 0.06-fold increase in ROS levels compared to the untreated group (*p* < 0.001), indicating the successful establishment of the oxidative stress model. The following data are presented as fold change to the H_2_O_2_-stimulated group.

In the dark, we found that ACVM significantly suppressed H_2_O_2_-induced ROS levels in a dose-dependent manner at 50 and 100 μg/mL. At both concentrations, the inhibitory effect of ACVM was comparable to that of the positive control (10 μg/mL l-ascorbic acid), with no statistically significant difference observed (Figure 7).

The antioxidant activity of ACV has mainly been evaluated using chemical assays. Yuan et al. reported that the ethyl acetate fraction of ACVM exhibited the strongest free radical scavenging activity at 852.5 μg/mL in the DPPH assay and 28.21 μg/mL in the ABTS assay (Yuan et al., 2012). Similarly, Rajurkar et al. found that the ethanol extract showed IC_50_ values of 398.6 μg/mL and 695 μg/mL in the DPPH and ABTS assays, respectively (Rajurkar and Gaikwad, 2012). Seif et al. further supported its antioxidant activity *in vivo*, by reporting that a 50% ethanol extract (200 mg/kg) suppressed carbendazim-induced increases in malondialdehyde (MDA) and H_2_O_2_ levels in mice (Seif et al., 2023). Moreover, an *in vitro* study revealed that a 59% ethanolic extract inhibited H_2_O_2_-induced lipid peroxidation and enhanced antioxidant enzyme activities (SOD, CAT, and GPx) in human lymphocytes, although the exact concentration used was not specified (Kumar, 2009). The present study is the first to report on its antioxidant activity in skin cells. Based on our HPTLC data, we suggest that the high rutin and chlorogenic acid content may account for the observed antioxidant effects after H_2_O_2_ challenge under dark conditions (Patel and Patel, 2019; Nguyen et al., 2024).

Treatment with ACVM and subsequent illumination further increased its antioxidant activity. At concentrations of 10 μg/mL and 50 μg/mL, ROS levels after light irradiation (0.76 ± 0.03-fold and 0.59 ± 0.02-fold, respectively) were significantly lower than those measured in the dark (0.90 ± 0.03-fold and 0.69 ± 0.10-fold, respectively). Notably, 10 μg/mL of l-ascorbic acid also led to a more pronounced reduction in ROS levels under light (0.52 ± 0.06-fold) compared to darkness (0.66 ± 0.09-fold). A possible mechanism is that l-ascorbic acid undergoes photodegradation, yielding ascorbyl radicals and dehydroascorbic acid (DHA) (Tikekar et al., 2011). DHA enters cells more efficiently than ascorbic acid through GLUT transporters and is quickly converted back into ascorbic acid within the cell, thereby boosting intracellular vitamin C levels (Wilson, 2002).

The enhanced antioxidant activity of ACVM after light exposure may be due to photochemical transformations of the active metabolites similar to those observed for ascorbic acid. However, it cannot be ruled out that the low-dose photodynamic conditions applied to the light-sensitive molecules in the extract led to the generation of small amounts of ROS. At such low levels, ROS rather function as signaling molecules that may trigger the upregulation of antioxidant defense systems (e.g., SOD, CAT, GPx) (Schieber and Chandel, 2014; Jomova et al., 2023). Interactions with other antioxidative molecules present in our extract, such *as* rutin and/or chlorogenic acid, may have also contributed to the higher net antioxidative effect observed under light exposure. However, the precise mechanisms underlying this enhanced antioxidant activity require further investigation.

On the other hand, it is well known that chlorogenic acid contains a catechol structure that may undergo redox reactions, while rutin, a polyhydroxylated flavonoid, may cause nonspecific adsorption. Both have been classified as borderline pan-assay interfering substances (PAINS). Therefore, their presence may potentially contribute to assay-dependent artefacts or false-positive results (Heinrich et al., 2020; Heinrich et al., 2022). To address this concern, we plan to perform a series of orthogonal validation experiments to exclude such possible interferences, including (1) ROS scavenger inhibition assays (e.g., NaN_3_, DABCO, catalase): to verify the causal role of ROS; (2) signal interference controls using cell-free systems; and (3) comparison between purified fractions and the original mixture using LC-MS isolation. These additional experiments will help to confirm that the observed effects indeed arise from genuine photodynamic activity rather than PAINS-like chemical artefacts (Heinrich et al., 2020). It is also worth noting that other antioxidants present in the ACVM extract, notably chlorophylls and their derivatives, may also contribute to this activity (Sun et al., 2024a).

### 3.7 Anti-inflammatory activity of ACVM

**FIGURE 8:**
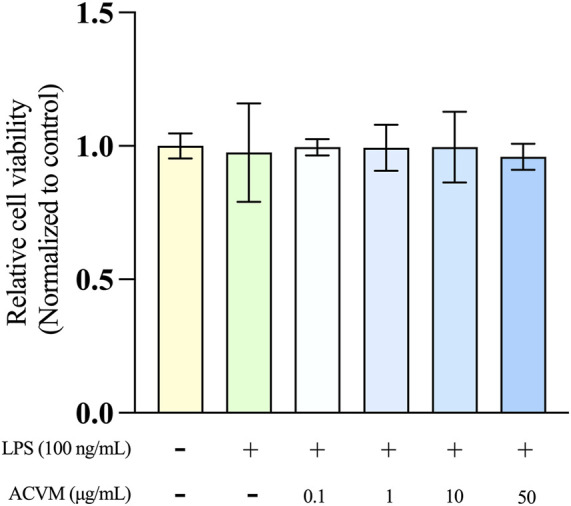
Effect of ACVM on the viability of LPS-stimulated RAW 264.7 cells after 24 h of incubation, assessed using the MTT assay. Results are expressed as the mean ± SD from three independent experiments. *p* < 0.05 (*), *p* < 0.01 (**) and *p* < 0.001 (***), indicates significant differences compared to the blank group. Data were normalized to the untreated blank group, which was set as 1 and presented as the fold change.

Since the scratch assays and DCFH-DA tests revealed that ACVM concentrations above 50 μg/mL (either under darkness or under visible-light exposure) impair cell migration-promoting and antioxidant effects, the maximum tested concentration for the anti-inflammatory assays was reduced to 50 μg/mL. Prior to conducting these studies, we confirmed that, for the chosen macrophage cell line (RAW264.7), neither the proinflammatory inducer LPS (100 ng/mL) alone, nor a combination of ACVM (0.1–50 μg/mL) and LPS (100 ng/mL) had a significant effect on cell viability, as evaluated with the MTT assay (Figure 8).

**FIGURE 9:**
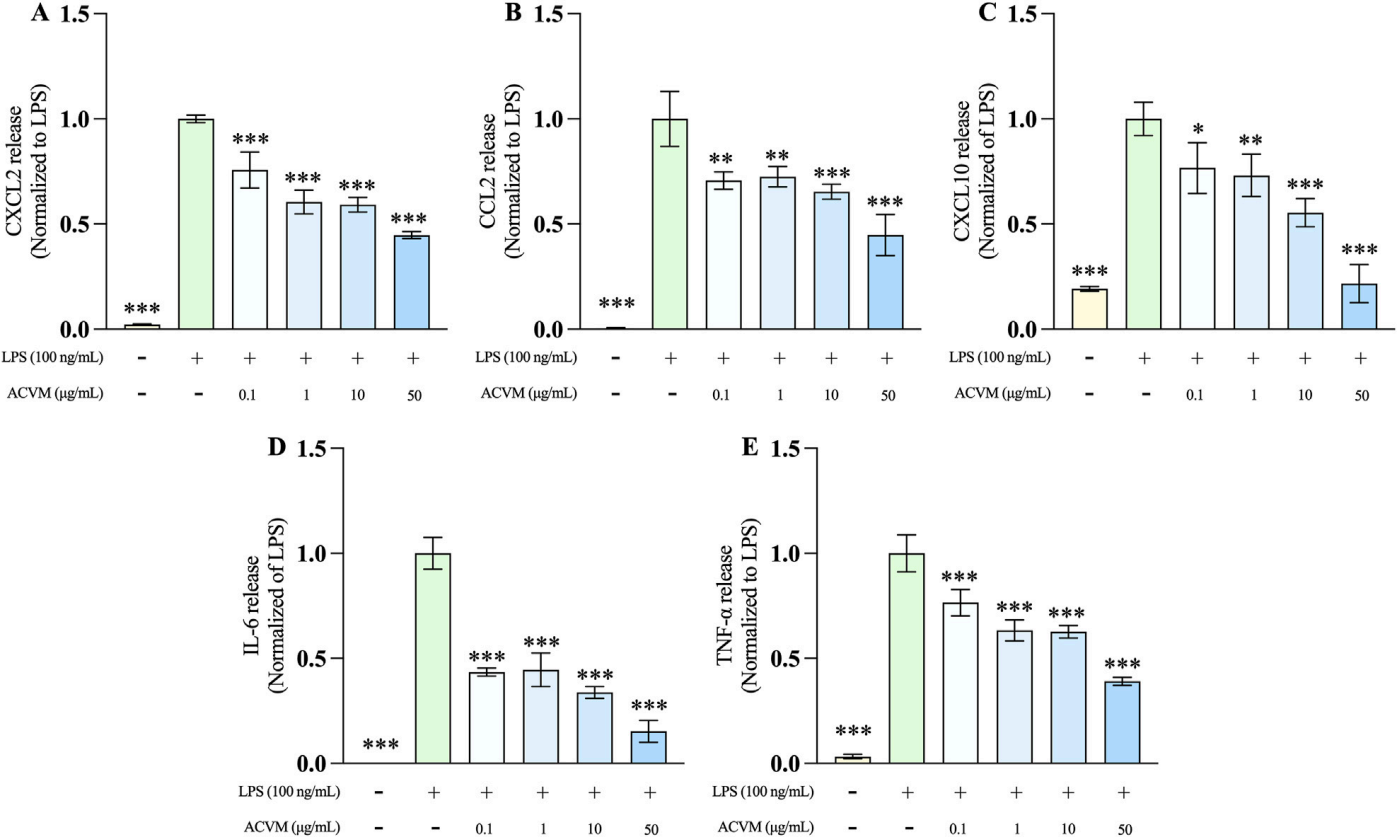
Effect of ACVM at different concentrations on LPS-induced release of CXCL2 **(A)**, CCL2 **(B)**, CXCL10 **(C)**, IL-6 **(D)**, and TNF-a **(E)** in RAW 264.7 cells. Results are expressed as mean ± SD from three independent experiments and normalized to the LPS group. *p* < 0.05 (*), *p* < 0.01 (**), and *p* < 0.001 (***) indicate significant differences compared to LPS group.

The effect of ACVM on LPS-induced production of three pro-inflammatory chemokines (CXCL2, CCL2, and CXCL10) and two cytokines (IL-6 and TNF-α) in RAW 264.7 macrophages was evaluated using ELISA. After 24 h of LPS stimulation, the secretion of all five inflammatory mediators increased significantly as expected, with levels exceeding a 5-fold rise relative to the untreated cells (Figure 9). ACVM significantly and dose-dependently inhibited the production of all measured cytokines and chemokines after LPS stimulation.

The anti-inflammatory activity of ACV has been demonstrated in multiple models. Aqueous and 80% ethanol extracts inhibited acetic acid-induced myeloperoxidase (MPO) production in rats at minimum effective doses of 300 mg/kg and 150 mg/kg, respectively (Khoramian et al., 2020). Consistently, ethyl acetate and methanol fractions, as well as isolated triterpenoids, significantly reduced carrageenan-induced edema and acetic acid–induced writhing, supporting both extract- and compound-level efficacy (Haider et al., 2011; Haider et al., 2013). Yuan et al. reported that ethanolic extracts suppressed LPS-induced PGE_2_ production in RAW 264.7 cells and IL-6 and TNF-α secretion in U937 monocytes, with IC_50_ values <50 μg/mL. Mechanistically, this effect was mediated by inhibition of NF-κB activation through reduced phosphorylation of IKKα/β, p38 MAPK, and p65 (Ser536) (Yuan et al., 2013).

This study further confirmed that the methanol extracts inhibited LPS-induced production of IL-6 and TNF-α in a dose-dependent manner. These two cytokines are most essential and commonly used inflammatory biomarkers serving as key nodes in multiple inflammatory signaling pathways (such as NF-κB, JAK/STAT, and MAPK) (Chen et al., 2017), reflecting both the intensity and progression of inflammation. Notably, for the first time, this study identified the inhibitory effect of the extract on the release of LPS-induced chemokines (CXCL2, CCL2, and CXCL10), which play a key role in immune cell recruitment and inflammation resolution (Borish and Steinke, 2003). Overexpression of CXCL10 engages its receptor CXCR3, leading to the inhibition of endothelial cell proliferation, suppression of angiogenic responses, and fibroblast activity (Rokni et al., 2025). Elevated levels of CXCL2 promote persistent macrophage infiltration, accompanied by increased TNF-α production, which sustains chronic inflammation and contributes to scar formation (Ridiandries et al., 2018). In contrast, CCL2 generally facilitates tissue repair. Impaired CCL2 expression delays macrophage recruitment and consequently hampers the wound healing process (Ishida et al., 2019).

As anti-inflammatory tests were performed in a collaborating institution without facilities for controlling light conditions, the experiments were carried out under standard ambient light, and no comparison of light-dependent effects was possible.

## 4 General discussion

This study investigated the wound-healing properties of ACV, combined with visible light phototherapy, as an innovative strategy. The findings suggest that the traditional use of ACV in wound healing is supported by its pharmacological activities, including pro-migratory, antioxidant, and anti-inflammatory effects. The introduction of visible light to the treatment protocol demonstrated significant potential for enhancing its wound healing properties.

Chemical analysis revealed the presence of key phytochemical chlorogenic acid, and the ability to absorb visible light, suggesting the potential of ACV for use in light-assisted therapy. However, the individual bioactivities of single compounds were not assessed in this study and are currently under investigation. Biological testing confirmed cytotoxicity and pro-migratory effects on keratinocytes, consistent with previous findings on dermal fibroblasts, thereby reinforcing the potential application of ACV in wound healing.

Some limitations should be acknowledged. Keratinocytes were the only skin cells used for the experiments. While keratinocytes are the primary cell type that forms the epidermal barrier, fibroblasts also play crucial roles in skin physiology and the wound-healing process. Including fibroblasts in future studies would help determine whether the observed effects are specific to keratinocytes, or represent a general cellular response in the skin. The scratch assay, although widely used and cost-effective for assessing cell migration, is sensitive to variability in wound gap uniformity due to operator technique (Kramer et al., 2013). To address this, future studies will employ complementary methods, such as the Boyden chamber assay, to assess and refine these findings. As the current anti-inflammatory assays did not include controlled light exposure, future work will incorporate defined light conditions, positive controls, and mechanistic analyses to more comprehensively assess the anti-inflammatory potential of ACVM.

This study demonstrated that light appears to enhance ACV extract activity at low concentrations. This effect diminished or even reversed at higher concentrations (e.g., 100 μg/mL). Only a single light condition was tested in this study, and the homogeneity of the light source requires further improvement. Hence, it remains possible that optimizing light parameters such as wavelength, intensity, or exposure duration could improve outcomes and reduce phototoxicity. Taken together, the light-dependent changes observed in this study may arise from two complementary mechanisms. The first mechanism involves intrinsic photobiomodulation, in which visible light influences cellular behavior by altering mitochondrial function, ATP production and the overall redox state (Hamblin, 2018), thereby supporting proliferation, migration and inflammatory resolution. The second mechanism relates to the interaction between light and photoactive constituents within the extract to generate ROS. At low extract concentrations, the controlled ROS production stimulate pro-healing signaling, including MAPK/ERK pathways that promote migration (Jang et al., 2013), and Nrf2 pathways that enhance antioxidant capacity, as well as temporary reductions in inflammatory pathways such as NF-κB and JAK STAT (Khorsandi et al., 2022).

Although the study focused on four wound healing-related activities, it raises broader questions about the extract’s potential in other relevant activities such as angiogenesis, extracellular matrix remodeling, and microbial defense. Whether light-assisted phototherapy is truly beneficial for wound healing depends on the results of further investigations into the combination of light and extracts on these biological activities. Targeted studies are needed to determine how light influences each process and whether its integration can reliably enhance therapeutic efficacy.

## 5 Conclusion

Collectively, our results demonstrate the feasibility of integrating plant-derived medicines with modern scientific technologies. This aligns with the goals of the WHO’s Traditional Medicine Strategy 2025–2034 by contributing rigorous, relevant research that could lead to more efficient and safer uses of medicinal plants (i.e., traditional medicine) and natural products in wound care. Although wound healing is one aspect of skin-related disorders, the bioactivities observed in this study may also be applicable to other dermatological conditions, suggesting a broader therapeutic potential for the investigated plant species. Moreover, these results reaffirm the remarkable potential of plant-based medicines in disease treatment. Moving forward, ensuring their safe and effective application and sustainable development remains a shared challenge among researchers and practitioners in this field, as well as a primary focus for our future work.

## Data Availability

The original contributions presented in the study are included in the article/Supplementary Material, further inquiries can be directed to the corresponding author.
