# Supplementary material for: Wound healing and photodynamic potential of Adiantum capillus-veneris L. (Pteridaceae): an evaluation of the cellular effects and molecular insights
Source: Front Pharmacol. 2026 Jan 5;16:1729572. doi: 10.3389/fphar.2025.1729572 (PMC12813077; doi:10.3389/fphar.2025.1729572)
Supplement: Supplementary file 1 [file Supplementaryfile1.docx]

Supplement Materials

Table 1 Chemical, solvents, buffer and medium information

| Chemicals/solvents/buffer/medium | Catalogue number | Company |
| --- | --- | --- |
| Chlorogenic acid | C3878 | Sigma-Aldrich, Gillingham, UK |
| Phosphate-buffered saline (PBS) | 10010-023 | Gibco, Waltham, USA |
| 2′,7′-dichlorodihydrofluorescein diacetate | 85155 | Cambridge Bioscience, Cambridge, UK |
| Acetone | 650501 | Sigma-Aldrich, Gillingham, UK |
| Dimethyl sulfoxide | A3672.0250 | AppliChem GmbH, Darmstadt, Germany |
| Dulbecco’s Modified Eagle Medium (DMEM) | 11594446 | Gibco, Waltham, USA |
| Epidermal growth factor | 50349.1 | Cambridge Bioscience, Cambridge, UK |
| Ethanol | 32221 | Sigma-Aldrich, Gillingham, UK |
| Ethyl acetate | 33211 | Sigma-Aldrich, Gillingham, UK |
| Fetal bovine serum (FBS) | 12484028 | Fisher Scientific, Loughborough, UK |
| Glacial acetic acid | A/0400/PB17 |  |
| Hydrogen peroxide | 10081254 |  |
| L-ascorbic acid | 255564 | Merck KGaA, Darmstadt, Germany |
| Methanol | 32213 | Sigma-Aldrich, Gillingham, UK |
| Mitomycin C | A4452-APE | Stratech Scientific Ltd., Ely, UK |
| Penicillin–streptomycin | 15140-122 | Gibco, Waltham, USA |
| Rutin | 102824 | MP Biomedicals, Solon, OH, USA |
| Sulforhodamine B sodium | A14769.06 | Alfa Aesar, Ward Hill, USA |
| Sulfuric acid | 1.60315 | Sigma-Aldrich, Gillingham, UK |
| Trichloroacetic acid | 91230 | Merck KGaA, Darmstadt, Germany |
| Toluene | T/2250/17 | Fisher Scientific, Loughborough, UK |
| Trizma base | T1503 | Sigma, St. Louis, UAS |
| Trypan blue solution | 10164110 | Fisher Scientific, Loughborough, UK |
| 1x TMB Solution | 50-112-9758 | Invitrogen, Carlsbad, USA |
| Trypsin–EDTA | T4049 | Merck KGaA, Darmstadt, Germany |
| Vanillin | V1104 | Sigma-Aldrich, Gillingham, UK |
| Water | 270733 |  |


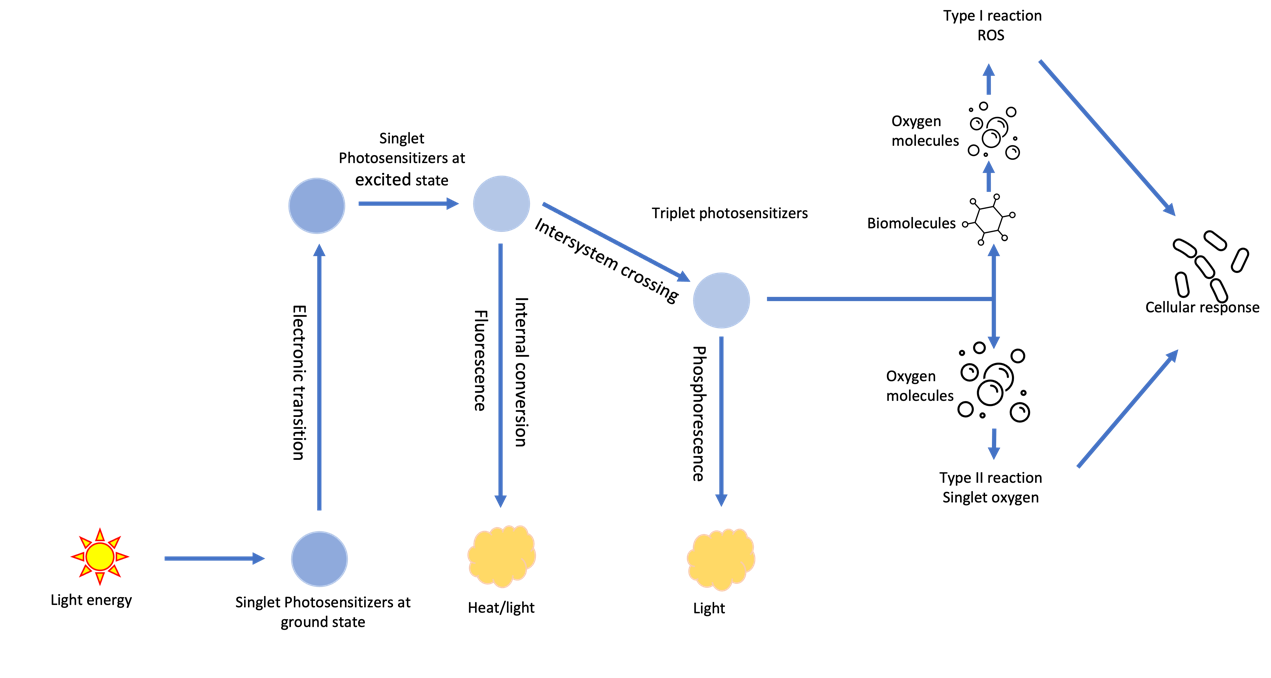


Figure 1 Modified schematic illustration of the photodynamic therapy mechanism (based on (Kwiatkowski et al., 2018))


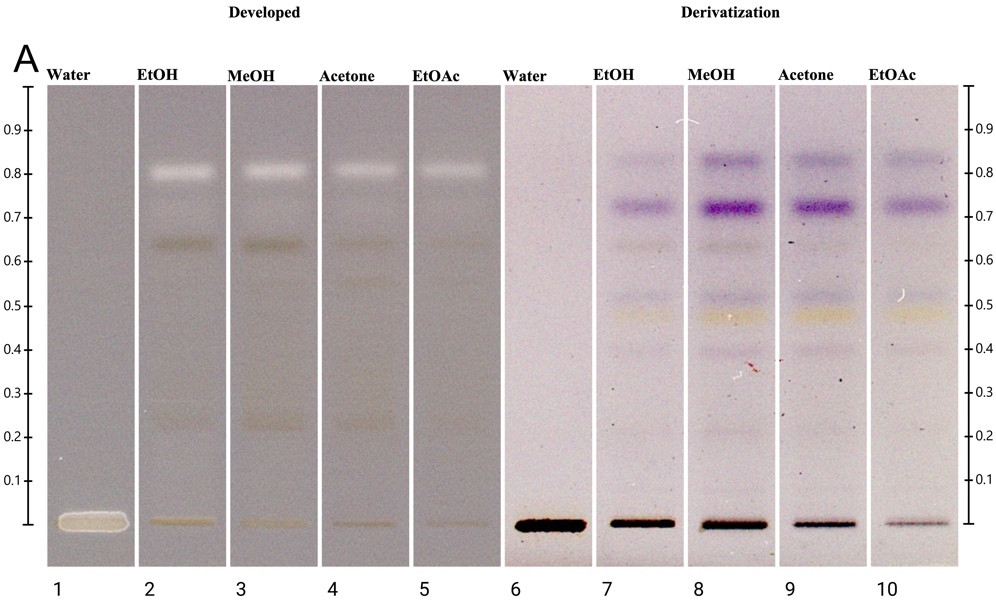


Figure 2 HPTLC profile of *A. capillus-veneris* extracts under white light visualization. The plate was first visualized without derivatization (labeled as “Developed”). Results after derivatization are labelled as “Derivatization” in this figure. All visualized bands are numbered according to the numbers assigned in Table 3.


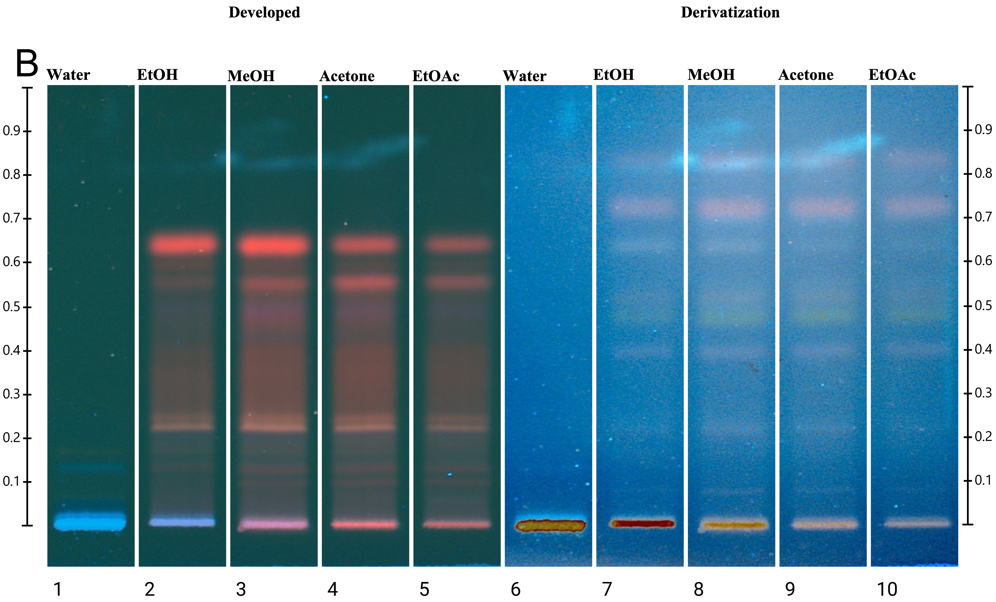


Figure 3 HPTLC profile of *A. capillus-veneris* extracts under 366 nm UV light visualization. The plate was first visualized without derivatization (labeled as “Developed”). Results after derivatization are labelled as “Derivatization” in this figure. Numbers X-Y highlight the additional bands that were not visible under the white light.


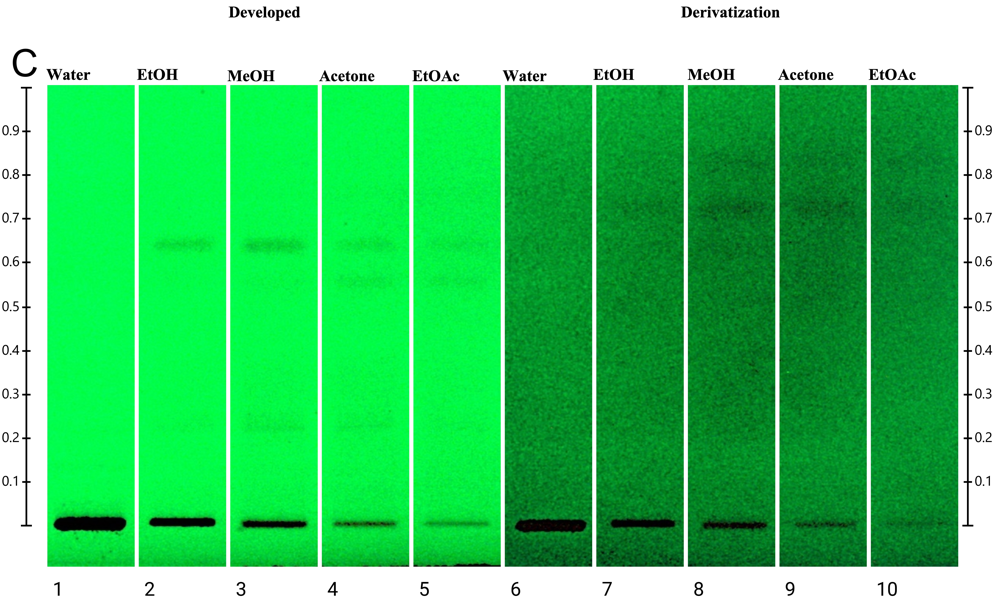


Figure 4 HPTLC profile of *A. capillus-veneris* extracts under white 254 nm UV light visualization. The plate was first visualized without derivatization (Labelled as Developed). Results after derivatization are labeled as Derivatization in this figure.

Reference List

Kwiatkowski, S., Knap, B., Przystupski, D., Saczko, J., Kędzierska, E., Knap-Czop, K., et al. (2018). Photodynamic therapy – mechanisms, photosensitizers and combinations. *Biomed. Pharmacother.* 106, 1098–1107. http://doi.org/10.1016/j.biopha.2018.07.049
